# Artificial Intelligence Can Effectively Predict Early Hematoma Expansion of Intracerebral Hemorrhage Analyzing Noncontrast Computed Tomography Image

**DOI:** 10.3389/fnagi.2021.632138

**Published:** 2021-05-26

**Authors:** Linyang Teng, Qianwei Ren, Pingye Zhang, Zhenzhou Wu, Wei Guo, Tianhua Ren

**Affiliations:** ^1^Department of Emergency, Beijing Tiantan Hospital, Capital Medical University, Beijing, China; ^2^Department of Functional Neurosurgery, Beijing Tiantan Hospital, Capital Medical University, Beijing, China; ^3^BioMind Technology, Beijing, China; ^4^Department of International Medical, Beijing Tiantan Hospital, Capital Medical University, Beijing, China

**Keywords:** predict, hematoma expansion, convolutional neural network, artificial intelligence, intracerebral hematoma

## Abstract

This study aims to develop and validate an artificial intelligence model based on deep learning to predict early hematoma enlargement (HE) in patients with intracerebral hemorrhage. A total of 1,899 noncontrast computed tomography (NCCT) images of cerebral hemorrhage patients were retrospectively analyzed to establish a predicting model and 1,117 to validate the model. And a total of 118 patients with intracerebral hemorrhage were selected based on inclusion and exclusion criteria so as to validate the value of the model for clinical prediction. The baseline noncontrast computed tomography images within 6 h of intracerebral hemorrhage onset and the second noncontrast computed tomography performed at 24 ± 3 h from the onset were used to evaluate the prediction of intracerebral hemorrhage growth. In validation dataset 1, the AUC was 0.778 (95% CI, 0.768–0.786), the sensitivity was 0.818 (95% CI, 0.790–0.843), and the specificity was 0.601 (95% CI, 0.565–0.632). In validation dataset 2, the AUC was 0.780 (95% CI, 0.761–0.798), the sensitivity was 0.732 (95% CI, 0.682–0.788), and the specificity was 0.709 (95% CI, 0.658–0.759). The sensitivity of intracerebral hemorrhage hematoma expansion as predicted by an artificial intelligence imaging system was 89.3%, with a specificity of 77.8%, a positive predictive value of 55.6%, a negative predictive value of 95.9%, and a Yoden index of 0.671, which were much higher than those based on the manually labeled noncontrast computed tomography signs. Compared with the existing prediction methods through computed tomographic angiography (CTA) image features and noncontrast computed tomography image features analysis, the artificial intelligence model has higher specificity and sensitivity in the prediction of early hematoma enlargement in patients with intracerebral hemorrhage.

## Introduction

Intracerebral hemorrhage refers to hemorrhage caused by the rupture of the blood vessels in the brain parenchyma, with the mortality rate as high as 40% and 54% after 1 month and 1 year of the rupture, respectively (Zia et al., 2009; Hansen et al., 2013; Poon et al., 2014). More than 40% of the patients with acute intracerebral hemorrhage developed secondary hematoma enlargement (Van Asch et al., 2010). Past studies have shown that hematoma enlargement is an independent risk factor for the poor prognosis of intracerebral hemorrhage (Davis et al., 2006; Dowlatshahi et al., 2011; Delcourt et al., 2012). However, it has also been proved that the limited hematoma expansion therapy without selection shows no significant improvement on the prognosis (mortality and disability rate). In fact, it may even increase the incidence of the related adverse events (Anderson et al., 2013; Qureshi et al., 2016). Therefore, it is extremely important to identify the risk of hematoma enlargement in time for future clinical intervention.

Noncontrast computed tomography (NCCT) plays an extremely important role in the diagnosis and treatment of hematoma enlargement of the intracerebral hemorrhage. Past studies indicate that computed tomographic angiography (CTA) spot sign is an independent risk factor for hematoma enlargement (Goldstein et al., 2007; Wada et al., 2007; Demchuk et al., 2012; Brouwers et al., 2015; Caplan, 2016). However, due to the high requirements of emergency CTA for patients and the burden on hospitals, there are significant limitations in the application of CTA for early detection. NCCT is easy to operate and is widely applied. The recent years have witnessed a surge in research on the prediction of hematoma enlargement with NCCT imaging markers, with the concepts of black hole sign, blend sign, CT hypodensities, island sign, and hematoma enlargement border proposed and validated for their clinical value in the prediction of hematoma enlargement (Lu et al., 2007; Ji et al., 2009; Boulouis et al., 2016; Li et al., 2017; Sporns et al., 2017; Yu et al., 2017). However, these markers have to be interpreted by trained doctors only because it is easy to be influenced by the readers’ experience and subjective judgment, and the related sensitivity is not high.

In recent years, segmentation methods based on deep learning have attracted increasing interest owing to their abilities of self-learning and generalization from large data volumes (Hosny et al., 2018; Wang et al., 2020). The application of artificial intelligence algorithm to further the intelligent analysis and mining of medical imaging information can better extract the image features in an image to obtain accurate internal correlation. In the entire analysis process, the interference of human subjective factors gets eliminated; as a result, the outcomes are more accurate and reproducible. In this study, we attempted to develop a prediction model of hematoma enlargement risk in patients with intracerebral hemorrhage based on the NCCT images so as to rapidly screen out the high-risk population of hematoma enlargement for preparing individualized clinical treatment and guidelines.

## Materials and Methods

### Data Preparation

In this study, the NCCT images of patients with spontaneous intracerebral hemorrhage from 84 public hospitals were collected for retrospective analysis between November 2011 and May 2018. Meanwhile, a retrospective study cohort of eligible patients with spontaneous intracerebral hemorrhage admitted to 26 public hospitals between April and October 2018 was collected. All NCCT scans were taken as a part of the routine nursing care. The patient inclusion criteria were diagnosis with spontaneous ICH and admission to the emergency department within 6 h from the symptom onset, with the availability of the baseline values and follow-up NCCT data within 48 h of the symptom onset. The patient exclusion criteria included secondary ICH, patients undergoing acute treatment with the potential to limit the ICH volume, and NCCT images of insufficient quality.

In this study, we initially included 3,016 patients, of which only approximately 20% experienced hematoma enlargement (HE). Several proportional HE cases (*n* = 1,183, 47.1%) were purposely accumulated in the retrospective dataset for use in the development of deep-learning systems. The retrospective dataset was randomly divided into the development set (*n* = 1,899, 75.5%) and validation dataset 1 (*n* = 615, 24.5%). Another retrospective dataset is validation dataset 2 (*n* = 502).

**Figure 1:**
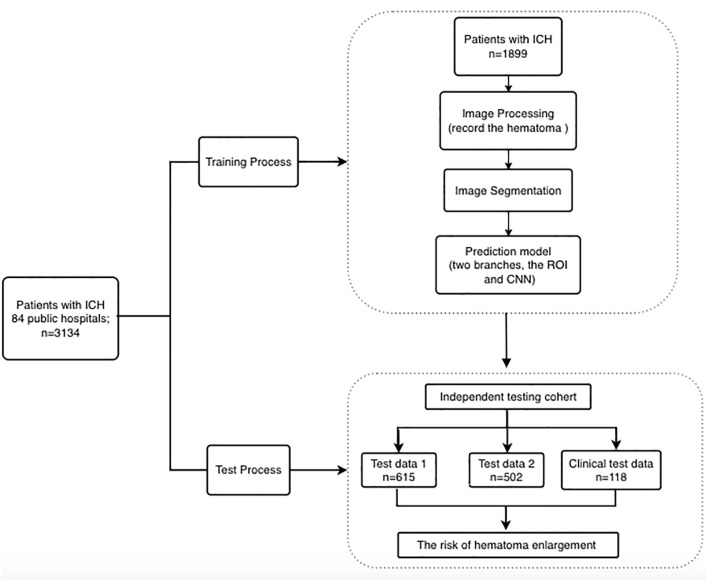
Flowchart of data preparation, image processing, system architecture, and evaluation.

For the clinical evaluation dataset, we retrospectively selected suitable patients from the Beijing Tiantan Hospital between June 2019 and April 2020 so as to assess the clinical behavior of the model. The inclusion criteria were patients diagnosed with spontaneous ICH and admitted to the Tiantan Hospital within 6 h from symptom onset, with the baseline images available, and who received a follow-up NCCT at 24 ± 3 h from the symptom onset. The exclusion criteria were secondary intracerebral hemorrhage that could not be recognized by the artificial intelligence model, patients treated with surgery within 24 h, and insufficient NCCT image quality. A total of 118 patients were finally involved in the dataset. Due to the sensitivity of the data used in the study, the data cannot be freely shared. We provided a flowchart of the process as Figure 1.

This study was conducted in accordance with the institutional review boards (IRBs) of Beijing Tiantan Hospital Affiliated to Capital Medical University, which also approved this study protocol. Patient information was anonymized and de-identified before the analysis.

### Image Processing

**Figure 2:**
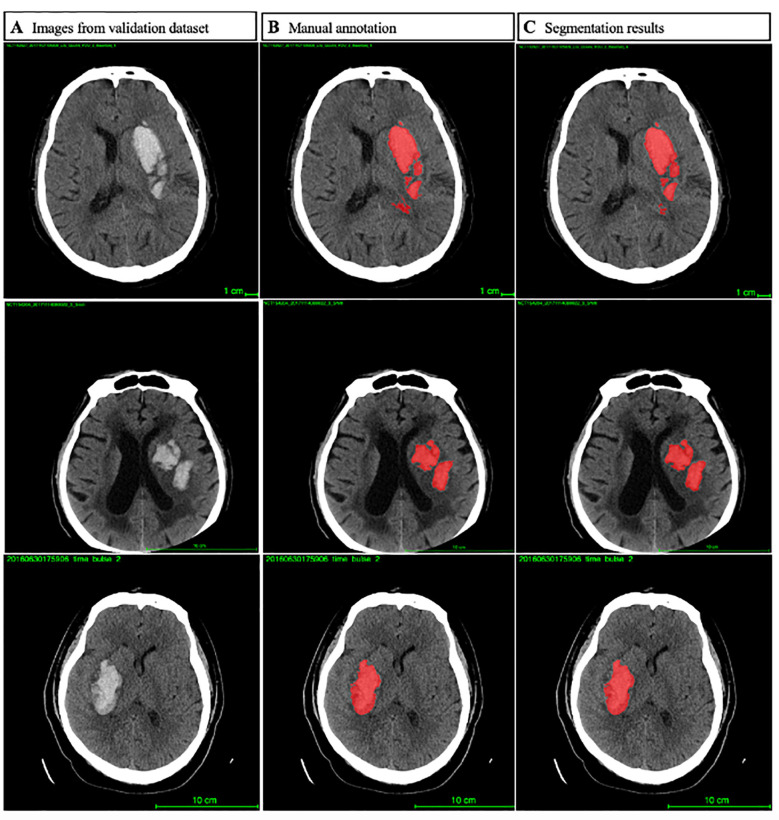
Image processing of the predict model.

In order to reduce potential human errors, we employed a hierarchical marking method for all scans in the database. No conflict of interest was registered among the doctors who participated in the project. Eight master’s degree candidates in neurology from the Beijing Tiantan Hospital received focused training for NCCT annotation and evaluation from a team of two experts (one neuroradiologist and one neurologist) with more than a decade of experience. The location and the presence of intracerebral hemorrhage were accordingly recorded as shown in Figure 2. For hematoma segmentation training and ICH volume calculation, each slice of hematoma appearing on the NCCT images was first drawn manually using the 3D Slicer analysis software by a trained rater, followed by independent reviewing by a second rater. The ICH volumes of the baseline and follow-up NCCT were calculated using a planimetric method. During the manual drawing phase, both intraparenchymal and intraventricular hemorrhage accounted for the same percent of hematoma volume. HE was defined as an increase in the hematoma volume of >6 ml (Dowlatshahi et al., 2011).

### System Architecture

#### Segmentation Model

**Figure 3:**
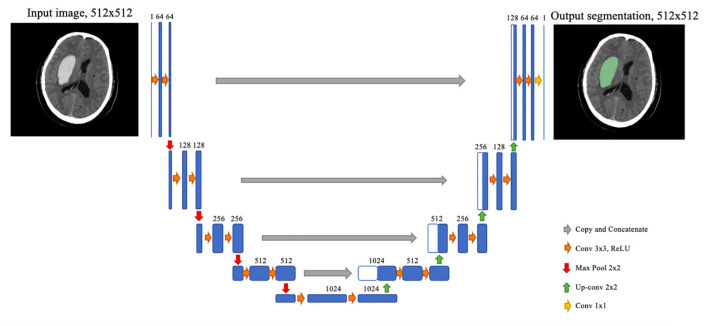
The running process of convolutional neural network applied in this study.

The segmentation model is based on a convolutional neural network termed U-Net, albeit with some modifications. Before feeding into the network, each slice of the original NCCT image was fit to a 512 × 512 square by cropping and padding if necessary, followed by normalization by a Hounsfield unit (HU) value window 0–100. The U-Net architecture consisted of two stages, the downsampling stage and the upsampling stage. Each block of the downsampling stage was composed of two 3 × 3 convolution layers and a 2 × 2 max-pooling layer. At the upsampling stage, the feature map from the last downsampling block was upsampled by a 2 × 2 up-convolution layer, concatenated with a feature map of the corresponding downsampling block, and then through two 3 × 3 convolution layers. After the final upsampling block, a 1 × 1 convolution layer with sigmoid activation was employed to determine the class of each pixel. To better catch the feature of hematoma, we used dilated convolution on the second convolution layers of each downsampling block (Figure 3).

#### Prediction Model

The inputs of the prediction model consisted of the original NCCT images of one patient and the segmentation results of the segmentation model that highlights the region of interest (ROI). The same inputs were propagated to the two branches of the prediction model.

The first branch generated radiomics features on the ROI. In this branch, image filters such as Laplacian of Gaussian and wavelet were applied on the images. Together with the original images, different classes of features including intensity, texture, and geometric were obtained on different image types. This process could yield 1379 quantitative features.

The other branch was a convolutional neural network (CNN) similar with the downsampling stage of U-Net, and a few repeated blocks consisted of convolution layers and a max pooling layer. The series of blocks terminates at a fully connected layer, and the final outputs were obtained from the last fully connected layer *via* softmax activation. Training was performed to optimize the prediction of the CNN, and the flattened output feature maps were extracted as the CNN features.

Finally, the radiomics features and the CNN features were concatenated and inputted to the gradient boosting classifier. Consequently, the outputs of the trained classifier were considered as the prediction results.

#### Clinical Evaluation

The purpose of the prediction system for hematoma enlargement of intracerebral hemorrhage based on NCCT scan is to rapidly, conveniently, and without human interference predict whether secondary hematoma enlargement can occur in patients with spontaneous intracerebral hemorrhage. After the patients with suspected cerebral hemorrhage completed their head NCCT scan, the conventional process NCCT images were uploaded to the hospital imaging system PACS by imaging physicians, and the images were synchronized to the AI imaging system; the images were automatically identified, and the interpretation results were output. In order to facilitate the clinical decision-making, the risk of hematoma enlargement was set according to the AUC curve analysis. If the risk value was higher than the threshold value and the patient was considered to possess secondary hematoma enlargement, the hematoma was considered stable if it was lower than the critical value. The Tiantan Hospital evaluated the clinical application value of BioMind, mainly from the specificity, the sensitivity, the positive predictive value, the negative predictive value, and the Yoden index of hematoma enlargement prediction. This article analyzed the causes of the operation errors of the specific case model.

### Statistical Analysis

All analyses were performed using SPSS software version 26.0 (IBM, Armonk, NY, USA). The AUC curve was used to evaluate the prediction results of BioMind, and the sensitivity and specificity were calculated. The count data were expressed in percentage (%). Continuous data were expressed either as medians and interquartile ranges or as mean ± standard deviation (SD). The demographic and clinical characteristics were compared among the patients with hematoma growth and without it using Student’s *t*-test or Mann–Whitney *U*-test, as appropriate. *P* < 0.05 was considered to be statistically significant.

## Results

### Model Performance

**Figure 4:**
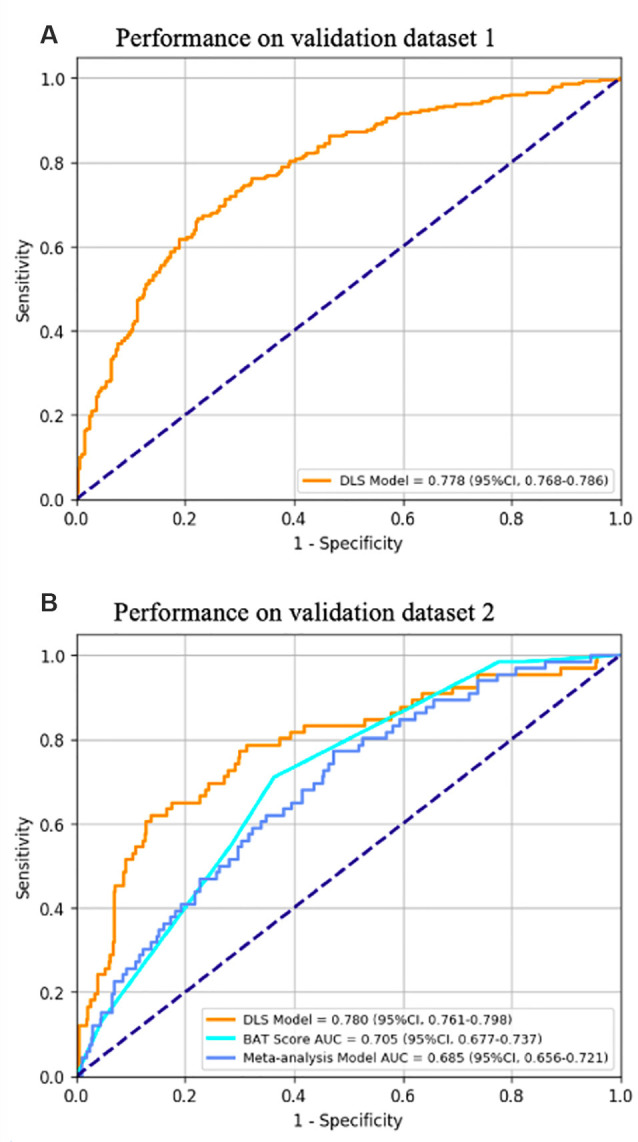
The performance of the HE prediction.

The DLS model produces a segmentation mask indicating the location of hematoma and a confidence score representing the risk of HE. For hematoma segmentation, in validation dataset 1 (*n* = 615, 15,980 images), the slice-level pixel-wise IoU was 0.863 (95% CI, 0.848–0.877), and the patient-level IoU was 0.831 (95% CI, 0.811–0.856). The performance of the HE prediction was measured by the area under the receiver operating characteristic curve (AUC). Figure 4 shows that in validation dataset 1, the AUC was 0.778 (95% CI, 0.768–0.786), the sensitivity was 0.818 (95% CI, 0.790–0.843), and the specificity was 0.601 (95% CI, 0.565–0.632). In validation dataset 2, the AUC was 0.780 (95% CI, 0.761–0.798), the sensitivity was 0.732 (95% CI, 0.682–0.788), and the specificity was 0.709 (95% CI, 0.658–0.759).

### Clinical Evaluation of BioMind

A total of 118 patients, including 84 (71.2%) male and 34 (28.8%) female, were enrolled in this study, with an average cohort age of 57.5 years. The basic characteristics of the patients included in the clinical validation datasets are summarized in Table 1. Among the 118 patients, 50 (42.4%) cases were with hematoma located in the basal ganglia, 26 (22.0%) cases in the thalamus, 30 (25.4%) cases in the lobe, and 12 (10.2%) in the cerebellum. Fifty-nine (50.0%) patients were found with an intraventricular hemorrhage on the baseline NCCT. Twenty-eight (23.7%) of the 118 patients suffered significant HE. We evaluated the ability of the model to predict hematoma enlargement in a clinical setting. In order to fit the clinical practice and facilitate the clinicians’ decision-making, the risk of hematoma enlargement is considered to be a critical value through the statistical analysis of the prediction efficiency of the model. The following outputs are defined as stable hematoma, and the abovementioned output results are hematoma enlargement. Through the cross-table analysis (Table 2), the sensitivity of intracerebral hemorrhage hematoma expansion predicted by the artificial intelligence imaging system was found to be 89.3%, with a specificity of 77.8%, a positive predictive value of 55.6%, a negative predictive value of 95.9%, and a Yoden index of 0.671.

**Table 1:** Comparison of the baseline demographic and clinical characteristics between patients with and without hematoma growth.

Variables	Hematoma growth (*n* = 28)	Without hematoma growth (*n* = 90)	*P*-value
Demographic			
Age, year (IQR)	48.5 (38.25–62.75)	60 (53–65.25)	0.012
Sex, male, *n* (%)	23 (82.1)	61 (67.8)	0.143
Medical history			
Hypertension, *n* (%)	18 (64.3)	64 (71.1)	0.493
Diabetes mellitus, *n* (%)	5 (17.9)	18 (20.0)	0.803
Coronary heart disease, *n* (%)	5 (17.9)	12 (13.3)	0.552
Alcohol consumption, *n* (%)	9 (32.1)	25 (27.8)	0.656
Smoking, *n* (%)	12 (42.9)	25 (27.8)	0.133
Clinical features			
Admission SBP, mmHg (median)	185.0	162.5	0.006
Admission DBP, mmHg (SD)	106.0 (21.2)	93.8 (16.2)	0.002
Baseline GCS score, median (mean rank)	8 (46.27)	9 (63.62)	0.018
Admission heart rate, median	96 (79.1)	78 (53.4)	<0.001
Location			0.117
Basal ganglia	17 (60.7)	33 (36.7)	
Thalamus	3 (10.7)	23 (25.6)	
Lobe	5 (17.9)	25 (27.8)	
Cerebellum	3 (10.7)	9 (10.0)	
Irregular hematoma	28 (100)	82 (91.1)	0.195
Intraventricular hemorrhage	9 (32.1)	50 (55.6)	0.030
Baseline ICH volume, ml (IQR)	18.3 (14.8–20.3)	13.9 (9.0–26.0)	0.310

*IQR, interquartile range; SBP, systolic blood pressure; DBP, diastolic blood pressure; SD, standard deviation; GCS, Glasgow Coma Scale; ICH, intracerebral hemorrhage*.

**Table 2:** Cross-table analysis of diagnostic value of BioMind.

	Actual Hematoma Growth *(n)*	Actual Hematoma Stable *(n)*	Total *(n)*
Predict Hematoma Growth *(n)*	25 (a)	17 (b)	42
Predict Hematoma Stable *(n)*	3 (c)	73 (d)	76
Total *(n)*	28	90	118

*Sensitivity: a/(a + c) × 100%, Specificity: d/(b + d) × 100%, positive predictive value: a/(a + b) × 100%, negative predictive value: d/(c + d) × 100%, Yoden Index: sensitivity + specificity −1*.

In addition, in the process of research, we found that the model cannot effectively operate in individual cases. The common feature of some cases is that the volume of hematoma is less than 3 ml, which leads to the result that the artificial intelligence model cannot judge the high-density shadow as the bleeding focus. Due to the small volume of the hematoma, the hematoma area on each slice is too small for the model to effectively distinguish the hematoma. The AI model also requires higher quality of images. Another part of patients may be anxious in the CT scanning process; in other words, they could not cooperate with the examination, resulting in lots of artifacts in the images. The strong interference made the model unable to identify and analyze effectively. These cases have been excluded in the data preparation.

## Discussion

In this study, an innovative deep-learning algorithm based on NCCT was performed and validated for the prediction of hematoma enlargement in patients with intracerebral hemorrhage. Our results showed that the deep-learning algorithm could automatically complete the hematoma labeling and analysis to rapidly produce accurate prediction results. This type of artificial intelligence analysis based on NCCT scan offers several advantages, including universality, safety, time effectiveness, independence of the experience level of the reader, high sensitivity, and high specificity.

Previous studies have proposed several imaging predictors to identify hematoma expansion. CTA spot sign, leakage sign, and spot-and-tail sign are some of the proven independent predictors of hematoma enlargement (Goldstein et al., 2007; Wada et al., 2007; Sorimachi et al., 2013; Caplan, 2016; Orito et al., 2016). However, all of the above imaging markers have a common shortcoming of requiring initial CTA examination. Several patients are not eligible for CTA examination during the early stages of the disease; the examination cost is high, and the examination is not popular as the first diagnosis especially in the emergency department. Even many institutions do not have the capacity to conduct CTA. The application of CTA as a primary screening test presents with significant limitations. NCCT scan is simple and easy to operate. As a common and necessary examination step for patients with acute cerebral hemorrhage, the sign of hematoma enlargement has gained a hot spot in recent studies. Research about NCCT image features in the prediction of hematoma enlargement has already achieved positive results. The blend sign, CT hypodensities, and island sign in NCCT images have been reported in succession related with hematoma enlargement (Boulouis et al., 2016; Li et al., 2017; Sporns et al., 2017) However, the disadvantage of these imaging markers is that they are all highly subjective. On one hand, their interpretation needs to be conducted manually, which takes a relatively long time and the results cannot be obtained immediately. On the other hand, the results of their interpretation are influenced by the doctors who read the images, such that the results in clinical practice show a huge deviation and are not stable.

Moreover, there may be more imaging signs and more complex connections between them, which are difficult to identify and summarize by naked eyes alone. At present, the methods based on the analysis of NCCT image features to predict the hematoma enlargement of intracerebral hemorrhage need to pick out the variables before the prediction analysis, that is, researchers need to identify the image features that may be related to hematoma enlargement. This process is likely to cause the loss of potential image features. But by the deep-learning model, we can directly import the processed images into the model without preset variables. The model can automatically learn and extract image features (Bengio et al., 2013; LeCun et al., 2015; Schmidhuber, 2015), greatly avoiding the loss of potential image feature information, so as to improve the specificity and sensitivity of prediction.

In addition, the deep-learning model and the convolutional neural network in image analysis have obvious strengths over other methods because of their own operational characteristics. Radiomics (Gillies et al., 2016; Lambin et al., 2017) is employed to extract a large number of high-dimensional quantitative imaging features from magnetic resonance imaging (MRI), positron emission tomography (PET), and NCCT images, which transform the conventional medical images into high-throughput imaging features that can be mined, quantitatively describe the spatial and temporal heterogeneity in the images, reveal the imaging features that cannot be recognized directly through the senses, effectively transform medical images into high-dimensional recognizable feature space, and analyze the generated spatial features with statistical analysis so as to establish models with predictive value that can provide worthy information for personalized diagnosis and treatment. The advantage of the deep convolution neural network is that it can automatically learn important low-level features (such as lines and edges) and can extract more complex and higher-level features (such as the shape) iteratively from low-level features (Fabijańska, 2018; Yasaka et al., 2018). Its end-to-end design provides more space for the model to automatically adjust according to the data and increase the overall fit of the model. Moreover, it retains the spatial relationship in filtering the input image, which can effectively extract the features of the images in the analysis of cerebral hemorrhage images. Moreover, the images are scaled and transformed in the training process, which greatly improves the stability of the output model.

Although our results are extremely promising, our study has some limitations. First, the learning process of the deep-learning model is known as a black box. Although we can develop and train the deep-learning model through data, we have no definite basis to explain the model. If the model predicts that a patient has a higher risk of early hematoma enlargement, we could not judge the imaging characteristics on which the prediction is based and could not explain the result. Second, only imaging data are included in the model at present, leaving the scope for including more statistically significant clinical features for comprehensive training through the analysis of clinical information of patients so as to further improve the clinical value of model prediction. Third, in this study, the predictive models did not automatically identify spontaneous intracerebral hemorrhage. There are some studies on the application of artificial intelligence to analyze the causes of cerebral hemorrhage with NCCT images with high accuracy. We believe that, if the two models can be combined, greater clinical benefits would be achieved. Fourth, this study was conducted with retrospective big data. As a result, we could not extract accurate past medical histories, such as the use of anticoagulants and antiplatelet drugs, which may also affect the risk of hematoma enlargement. We will conduct a prospective study in the next step to validate the artificial intelligence model and explore the correlation between medical histories, drug use, and hematoma enlargement.

In conclusion, BioMind is a valuable hematoma expansion prediction system. As compared with existing hematoma prediction methods, it provides a time-saving, easy to implement, and subjective independent method to predict the risk of hematoma enlargement in patients with intracerebral hemorrhage. Presently, BioMind has realized the software transformation, and the artificial intelligence imaging analysis can be realized after installation to become a part of the diagnosis and treatment process of cerebral hemorrhage for more customized diagnosis and treatment of patients with cerebral hemorrhage.

## Data Availability Statement

The raw data supporting the conclusions of this article will be made available by the authors, without undue reservation.

## Ethics Statement

The studies involving human participants were reviewed and approved by IRB of Beijing Tiantan Hospital Affiliated to Capital Medical University. The patients/participants provided their written informed consent to participate in this study.

## Author Contributions

The studies were conceptualized, results analyzed, and manuscript drafted by LT and QR. PZ wrote the code, trained the models, and wrote the first draft of the manuscript, with guidance from TR. Also, PZ and ZW provided raw training data and overall study supervision. WG provided supervision of clinical data collection. All authors contributed to the article and approved the submitted version.

## Conflict of Interest

Authors PZ and ZW were employed by company BioMind Technology. The remaining authors declare that the research was conducted in the absence of any commercial or financial relationships that could be construed as a potential conflict of interest.
